# Identifying phage Lysins through genomic analysis of prophages from *Acinetobacter baumannii*

**DOI:** 10.3389/fmicb.2025.1532950

**Published:** 2025-04-01

**Authors:** Maria Leonor Raposo, Ana Carolina Pimentel, Vera Manageiro, Aida Duarte, Manuela Caniça, Filipa F. Vale

**Affiliations:** ^1^Faculdade de Ciências, BioISI – Instituto de Biossistemas e Ciências Integrativas, Universidade de Lisboa, Lisbon, Portugal; ^2^Faculty of Pharmacy, Research Institute for Medicines (iMed-ULisboa), Universidade de Lisboa, Lisbon, Portugal; ^3^National Reference Laboratory of Antibiotic Resistances and Healthcare Associated Infections, Department of Infectious Diseases, National Institute of Health Dr. Ricardo Jorge, Lisbon, Portugal; ^4^Centre for the Studies of Animal Science, Institute of Agrarian and Agri-Food Sciences and Technologies, University of Porto, Porto, Portugal; ^5^AL4AnimalS, Associate Laboratory for Animal and Veterinary Sciences, Lisbon, Portugal; ^6^Faculty of Pharmacy, Universidade de Lisboa, Lisbon, Portugal; ^7^Egas Moniz Center for Interdisciplinary Research, Egas Moniz School of Health and Science, Almada, Portugal; ^8^CIISA, Center for Interdisciplinary Research in Animal Health, Faculty of Veterinary Medicine, University of Lisbon, Lisbon, Portugal

**Keywords:** *Acinetobacter baumannii*, antibiotic resistance, prophages, lysins, phylogeny

## Abstract

*Acinetobacter baumannii* is a Gram-negative opportunistic pathogen, responsible for nosocomial infections worldwide. In recent years, this microorganism has acquired resistance to various antibiotics, prompting the World Health Organization (WHO) to declare carbapenem-resistant *A. baumannii* (CRAB) a critical priority microorganism requiring urgent attention and the development of new therapeutic options. Here, we screened for prophages in 158 genomes of *A. baumannii*, comprising 139 complete genomes from the Bacterial and Viral Bioinformatics Resource Center (BV-BRC), and 19 newly sequenced clinical isolates. Additionally, we conducted phylogenetic analyses of prophages, highlighting their diversity and local clustering. The analyzed genomes harbored at least two prophage regions, resulting in the identification of a total of 950 prophage regions, of which 348 were considered complete prophages through software analysis and manual curation, while the remainder may represent prophage remnants. The complete prophages ranged from 28.6 to 103.9 kbp, with an average GC content of 39%. Based on genomic similarity, only 18 complete prophages were taxonomically classified to the genus *Vieuvirus*. Among all identified complete prophages, we identified 166 genes encoding for putative lysins, while prophage regions that were not considered complete could also harbor putative lysins. These findings highlight the abundance of prophage-encoded lysins in *A. baumannii* genomes, which are promising therapeutic agents for combating *A. baumannii* infections, particularly in the face of rising antibiotic resistance.

## 1 Introduction

Bacterial infections significantly affect human health and are a leading cause of morbidity and mortality worldwide. The emergence of multi-drug resistant (MDR) bacterial pathogens, such as the ESKAPE group (*Enterococcus faecium*, *Staphylococcus aureus*, *Klebsiella pneumoniae*, *Acinetobacter baumannii*, *Pseudomonas aeruginosa*, and *Enterobacter* spp.), exacerbates the current situation (De Oliveira et al., 2020). Among these, *A. baumannii*, an opportunistic, strictly aerobic, catalase-positive, oxidase-negative, and non-fermentative Gram-negative pathogen in the *Moraxellaceae* family, poses a significant threat as a primary cause of hospital-acquired infections and, less frequently, community-acquired pneumonia (Dexter et al., 2015). Common nosocomial infections include pneumonia, septicemia, urinary tract infections, skin and wound infections, endocarditis, and meningitis (Basatian-Tashkan et al., 2020), with ventilator-associated pneumonia (VAP), and bloodstream infections leading in mortality rates (Antunes et al., 2014).

In 2017 and again in the 2024 revision, the World Health Organization (WHO) designated carbapenem-resistant *Acinetobacter baumannii* (CRAB) as a priority pathogen for new antibiotic development (World Health Organization, 2017, 2024). Currently, *A. baumannii* exhibits resistance not only to carbapenems but also to last-resort antibiotics such as colistin (Cai et al., 2012) and tigecycline (Garnacho-Montero and Amaya-Villar, 2010). *A. baumannii* strains can naturally acquire exogenous DNA through natural transformation and plasmids via conjugation, contributing to the acquisition of antibiotic resistance genes (Domingues et al., 2019; Salgado-Camargo et al., 2020). Prophages harbor potential virulence factors, fitness-related genes, and antibiotic resistance genes. Prophages are major mediators of horizontal gene transfer, promoting bacterial genetic diversity (Costa et al., 2018).

Considering the increasing incidence of MDR bacterial infections, there is an urgent need to explore new alternatives to antibiotic administration. Given these challenges, exploring the use of bacteriophages, and their encoded lysins, is essential for developing novel antimicrobial strategies. Bacteriophages are viruses that specifically infect bacteria to ensure their survival and replication and exhibit two main types of replication cycles. The lytic cycle starts with viral particle attachment to the bacterial cell surface and the injection of viral DNA. Using the host machinery, viral DNA is amplified, and phage proteins are synthesized and assembled into a capsid. Finally, the cell lyses, releasing new virions (Ge et al., 2020). In the lysogenic cycle, the viral genetic material integrates into the bacterial genome, forming a prophage. This integration can be temporary, allowing the prophage to excise and reenter the lytic cycle (Ghose and Euler, 2020).

Recent studies of prophages have focused on identifying sequences encoding lysins that degrade the peptidoglycan layer, leading to cell lysis. Virion-associated lysins are involved in the injection of the phage genetic material into the cell. On the other hand, endolysins are expressed at the end of the phage replication cycle and promote the release of viral progeny by lysing the cell from within (Schmelcher et al., 2012). Phage lytic enzymes cleave the peptidoglycan layer and are classified as glycosidases, amidases, or endopeptidases based on the bonds they break (Danis-Wlodarczyk et al., 2021). External application of endolysins to Gram-positive bacteria exerts a lethal effect, as the exposed peptidoglycan allows these enzymes to access their target (Schmelcher et al., 2012). However, Gram-negative bacteria display an outer membrane that acts as a selective barrier (Masi and Pagès, 2013). The activity of lysins against Gram-negative bacteria can be enhanced in the presence of membrane-destabilizing factors, such as changes in pH or temperature, removal of Ca^2+^ or Mg^2+^, or the addition of ethylenediaminetetraacetic acid (EDTA) (Dillon, 2014). These observations have prompted the engineering of lysins by fusing them with peptides that destabilize the outer membrane for access to peptidoglycan. Nonetheless, several phage lysins with intrinsic antibacterial activity against various Gram-negative bacteria, including *A. baumannii*, have also been described (Sykilinda et al., 2018; Chu et al., 2022).

The aim of this study was to investigate endolysins encoded by prophages in selected *A. baumannii* genomes. For that, we conducted a comprehensive search of prophages in *A. baumannii* genomes, followed by the identification of endolysins encoded in these prophages, with a particular focus on complete prophages. By elucidating the potential mechanisms of action of these endolysins based on their annotation, this research aims to identify specific candidates for further investigation, thereby contributing to efforts to address the challenge of antimicrobial resistance.

## 2 Materials and methods

### 2.1 *Acinetobacter baumannii* genomes

A total of 139 *A. baumannii* genomes were selected from the Bacterial and Viral Bioinformatics Resource Center (BV-BRC).^1^ The selected genomes met the following criteria: host - *Homo sapiens*, genome quality - good, and genome status - complete. Some of the genomes of this set had previously been analyzed by Loh et al. (2020) and Costa et al. (2018).

Additionally, 9 genomes from *A. baumannii* isolates provided by the Faculty of Pharmacy of the University of Lisbon (FFUL) and 10 genomes from the National Health Institute Dr. Ricardo Jorge (INSA) were sequenced. After genomic DNA extraction, using QIAamp DNA Mini kit (Qiagen, United Kingdom) according to the manufacturer’s instructions, DNA yield and integrity were assessed using a Qubit assay and agarose gel electrophoresis. High-quality samples were used to prepare Nextera XT Illumina paired-end libraries, which were sequenced (2 × 150 bp) on the Illumina MiSeq platform, following the manufacturer’s instructions. Bacterial genomes were *de novo* assembled using the SPAdes 3.13 algorithm (Prjibelski et al., 2020). The inclusion of these additional genomes was important to enhance the genetic diversity represented in our analysis, providing a more comprehensive view of the genomic landscape of *A. baumannii* prophages. We incorporated locally sourced genomes to explore whether the prophages within these genomes share similarities with those from other regions, assessing the potential implications for localized versus generalized applications in phage-based therapies.

### 2.2 Prophage regions identification

All *A. baumannii* genomes were annotated by Rapid Annotation using Subsystem Technology (RAST) (Aziz et al., 2008) and screened to identify possible prophage regions using the open-access server PHAge Search Tool Enhanced Release (PHASTER) (Arndt et al., 2016) and PhiSpy 4.1.20 (Akhter et al., 2012). PHASTER, a database-based tool, classifies prophages as intact, questionable, or incomplete; intact prophages contained all essential genes for phage functionality, questionable ones had partial gene content, and incomplete prophages lacked many key structural and replication genes. PhiSpy detection is based on key genomic features and phage-specific patterns, but does not group them. We have considered as complete prophages only those that were identified by both programs, excluding the prophages classified as incomplete or questionable by PHASTER. Additionally, to pass the criteria of complete prophages, at least an integrase and a phage structural gene, namely: major capsid, minor capsid, major tail, minor tail, tape measure protein, baseplate protein, portal protein, among others that were related to the structure, should be present. Next, the complete prophages were manually curated to discern their boundaries. All prophage regions that were not considered complete are hereinafter referred as uncertain prophages.

To delimit these regions accurately, the amino acid sequences of the genes in the terminal prophage regions were compared using the Basic Local Alignment Search Tool (BLASTp), enabling the identification of similarities and functional relationships with known protein sequences. Additionally, MegaBlast was utilized to define the prophage sequences by identifying the bacterial genes flanking the prophage regions, thus refining the boundaries of the identified prophages.

CheckV v1.0.3 (Nayfach et al., 2021), a fully automated command-line pipeline for assessing the quality of single-contig viral genomes, was employed to evaluate the completeness and quality of the identified prophage sequences. CheckV generates a report file and assigns query contigs to one of five quality tiers: complete, high-quality (>90% completeness), medium-quality (50–90% completeness), low-quality (<50% completeness) and undetermined quality.

### 2.3 Phylogenetic analysis of prophages

The complete prophage sequences were aligned using the standard option adjust direction of Multiple Alignment using Fast Fourier Transform (MAFFT) version 7 (Katoh and Standley, 2013), and a maximum likelihood phylogenetic tree was constructed based on the nucleotide alignment, using the FastTreeMP 2.1.11 tool (Price et al., 2010). The visualization and annotation of the tree were improved using the Interactive Tree Of Life (iTOL) v4 tool (Letunic and Bork, 2019).

Additionally, to assess the phylogenetic relationships of the complete prophages within a broader viral taxonomy context, we used ViPTree: the Viral Proteomic Tree server version 4.0 (Nishimura et al., 2017). ViPTree generates proteomic trees based on genome-wide sequence similarities computed by tBLASTx, allowing for comprehensive visualization of viral genome relationships.

### 2.4 Taxonomic classification of complete prophages

Each complete prophage sequence was uploaded in the Webversion of the program taxMyPhage (Millard et al., 2024). This tool provides taxonomy at the genus or species level for a predicted phage. The output consists of an upper right matrix of similarity against other phages classified by the International Committee on the Taxonomy of Viruses (ICTV) and the assigned taxonomy.

### 2.5 Putative lysins identification

Complete prophages were analyzed for the presence of proteins possibly related to lysis. For this purpose, annotations provided by PHASTER and RAST were analyzed using the following keywords: “endolysin,” “lysin,” “lysozyme,” “lysis protein,” “hydrolase,” “transglycosylase,” “glycosidase,” “amidase,” “peptidase,” “protease,” “proteinase,” “lipase,” “tail lysozyme,” “endopeptidase” and “hypothetical protein” (the latter considered only in the two positions upstream and downstream of the holin-coding gene). The identified proteins were analyzed for sequence similarity by BLASTp, against non-redundant protein sequences and specific databases: bacteriophages (taxid: 38018), phage sp. (taxid: 38018), *Myoviridae* sp. (taxid: 2202564), *Siphoviridae* sp. (taxid: 2170413) and *Podoviridae* sp. (taxid: 2202567).

The proteins whose BLASTp results showed that they were related to cell wall lysis remained under study and were named putative lysins. We used Phyre2 to predict protein structure and identify structurally similar proteins, aiding in the determination of the putative function (Kelley et al., 2015). Prophage regions detected and putative lysins identified were manually annotated in the respective genomes using the bioinformatics tool Geneious Prime 2020.1.1.^2^

## 3 Results

### 3.1 *Acinetobacter baumannii* genomes

The average size of the 139 genomes obtained from BV-BRC was 4,003,639 bp (±127,085). *De novo* sequenced genomes had an average size of 3,948,248 bp (±103,545). Considering all the genomes analyzed, the average size was 3,998,437 bp (± 127,578). The average GC content across all genome sets was 39%.

### 3.2 Identification of prophage regions in *Acinetobacter baumannii* genomes

According to PHASTER, the 158 genomes carried prophages, totaling 878 prophage regions, including 406 intact (46.24%), 107 questionable (12.19%), and 365 incomplete (41.57%) prophages. Concerning PhiSpy, there were 628 prophage regions in 154 genomes.

**Figure 1:**
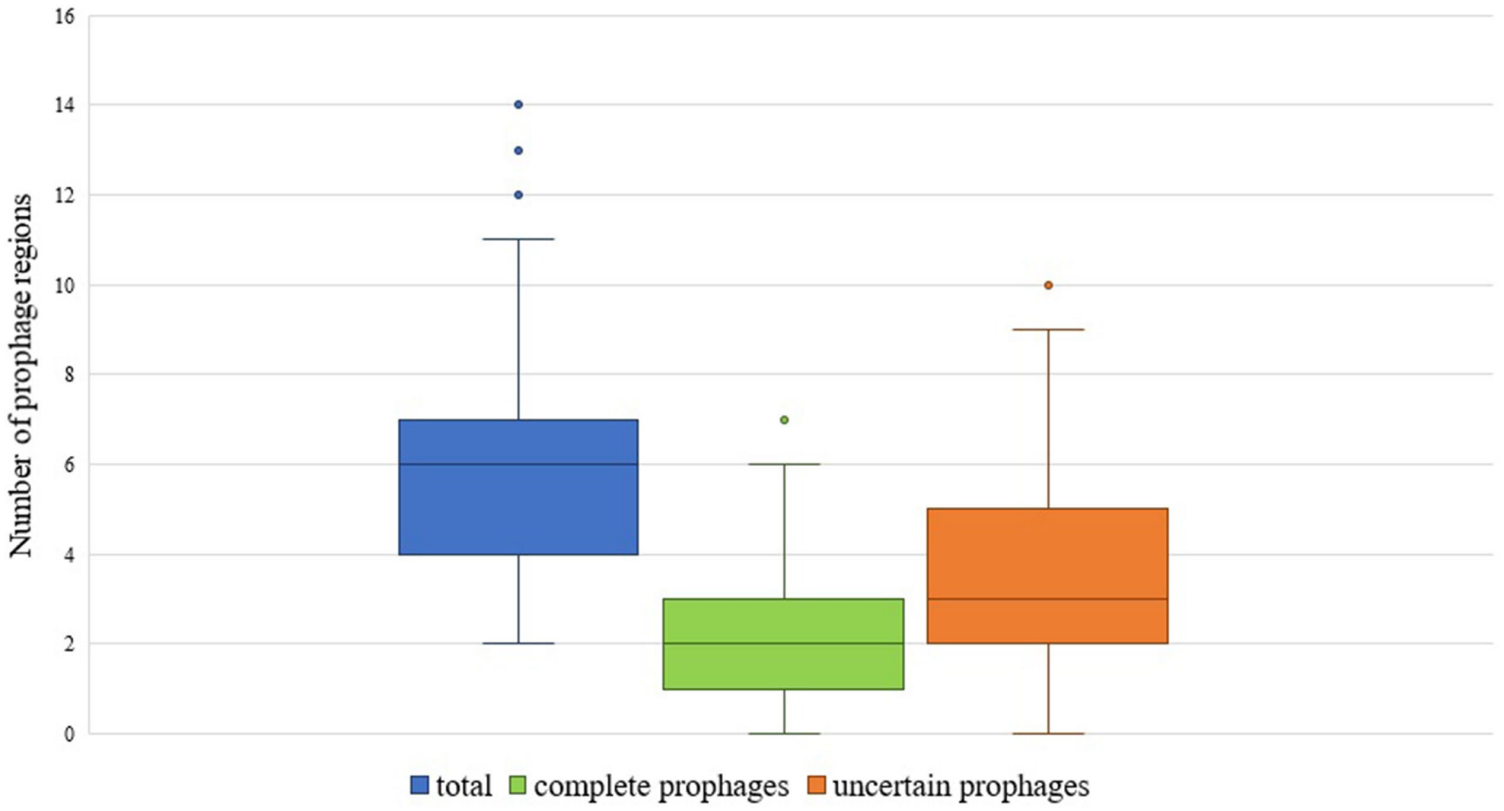
Box plot showing the number of total, complete and uncertain prophages in *A. baumannii* genomes.

**Figure 2:**
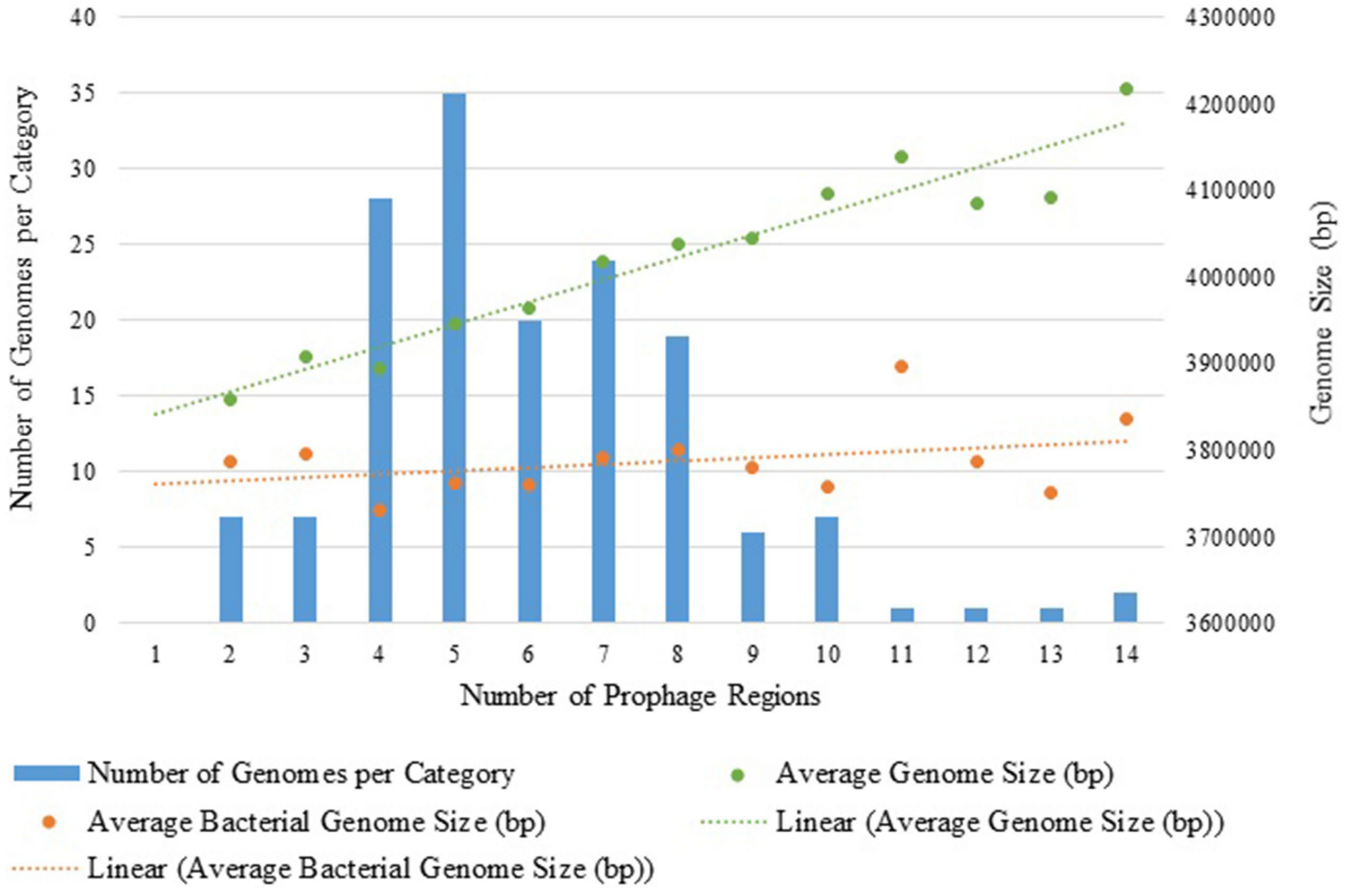
Distribution of prophage regions in 158 *A. baumannii* genomes: bar graph of genome counts and dots representing average genome size across prophage categories with linear regression analysis. The green line represents the average genome size (bacterial genetic material and prophage regions) for each category, while the orange line indicates only the bacterial core genome size.

The total number of prophage regions per genome ranged from 2 to 14, with an average of 6.01 (±2.29) prophages per genome, and most genomes harbored five prophage regions (n = 35) (Figures 1, 2). The maximum number of prophage regions found in a single strain genome was 14 (strains AB030, CP009257; Ab-D10a-a, CP051869).

We explored whether larger *A. baumannii* genomes harbored more prophage regions than smaller ones (Figure 2). To accomplish this, the number of prophage regions was analyzed in relation to the total genome size and the bacterial genome excluding prophages. It was observed that the increase in genome size was primarily due to the insertion of prophages, not other mobile elements, as the core bacterial genome remained constant. In agreement, a linear regression analysis showed an R^2^ of 0.114 for the relationship between the bacterial genome size excluding the prophage regions and the number of prophages, indicating that the bacterial genome size excluding prophages remains stable. In contrast, the regression of total genome size versus the number of prophage regions yielded an *R*^2^ of 0.921, suggesting that prophage regions significantly contribute to genome expansion, though not exclusively.

### 3.3 *Acinetobacter baumannii* complete prophages

A total of 348 complete prophages were identified in 143 bacterial genomes, with an average size after delimitation of 48.605 bp (±13.777). These prophage regions contained a significant number of genes coding for hypothetical proteins and several domains of unknown function that have not been explored. The smallest prophage genome was 28,620 bp and the largest 103,938 bp. The average GC content was 39%.

The quality assessment of the identified prophage sequences using CheckV revealed that 259 sequences (74.4%) were classified as high-quality, while 89 (25.6%) were categorized as medium-quality. This step was crucial to ensure that subsequent taxonomic and evolutionary analyses were based on high-confidence sequences.

**Figure 3:**
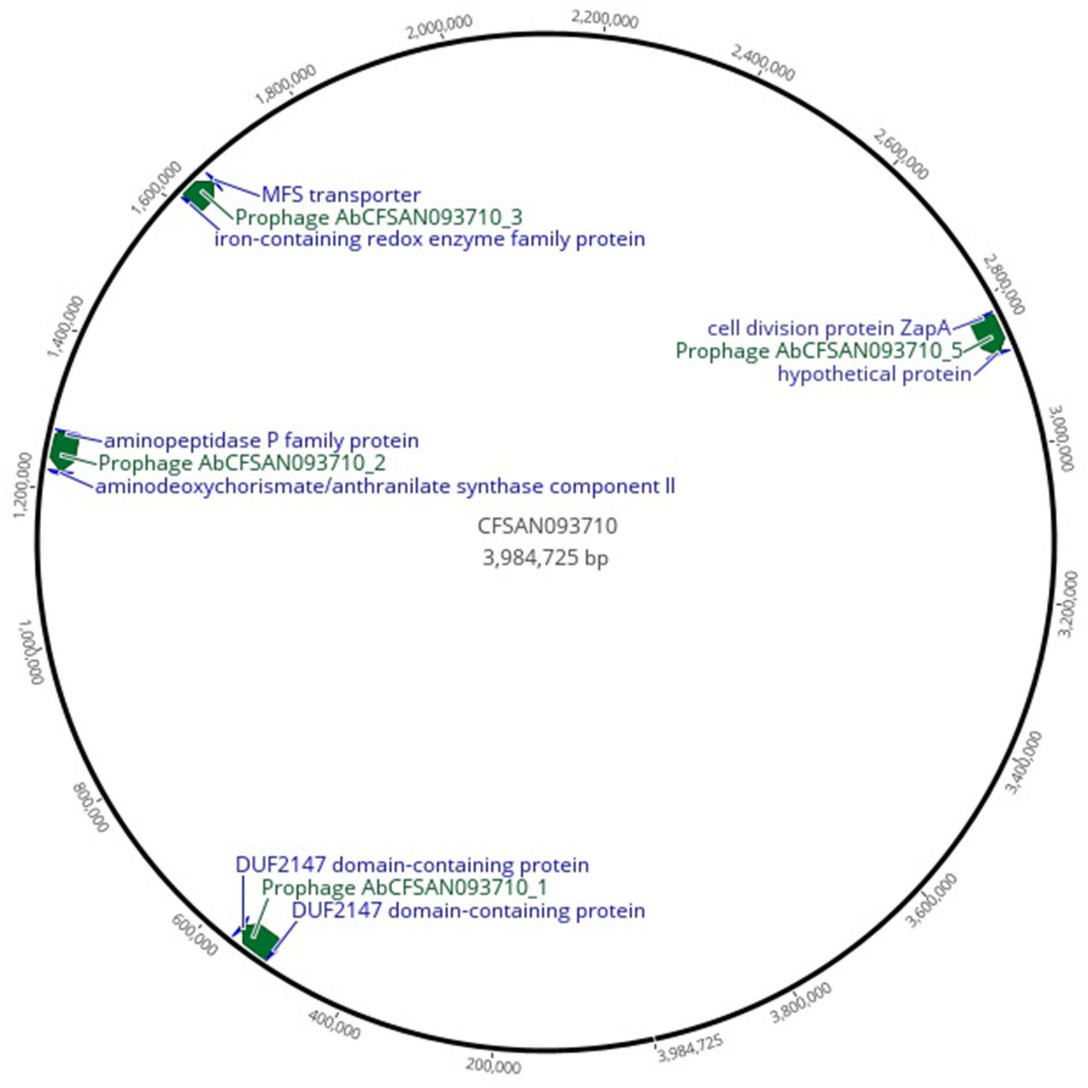
Genome map highlighting complete prophages distribution and adjacent bacterial genes that are hotspots for prophage integration. The figure was produced using Geneious Prime, 2025.0.3 (https://www.geneious.com; Geneious Prime, 2025).

The complete prophages presented hotspots for integration (Figure 3; Supplementary Table S1), with the top three integrations occurring adjacent to the bacterial genes: *ssrS* (6S RNA) and a hypothetical protein; iron-containing redox enzyme family protein and Major Facilitator Superfamily (MFS) transporter; and aminopeptidase P family protein and aminodeoxychorismate/anthranilate synthase component II. Additionally, numerous prophages integrate adjacent to tRNA genes.

### 3.4 Classification of *Acinetobacter baumannii* complete prophages

**Figure 4:**
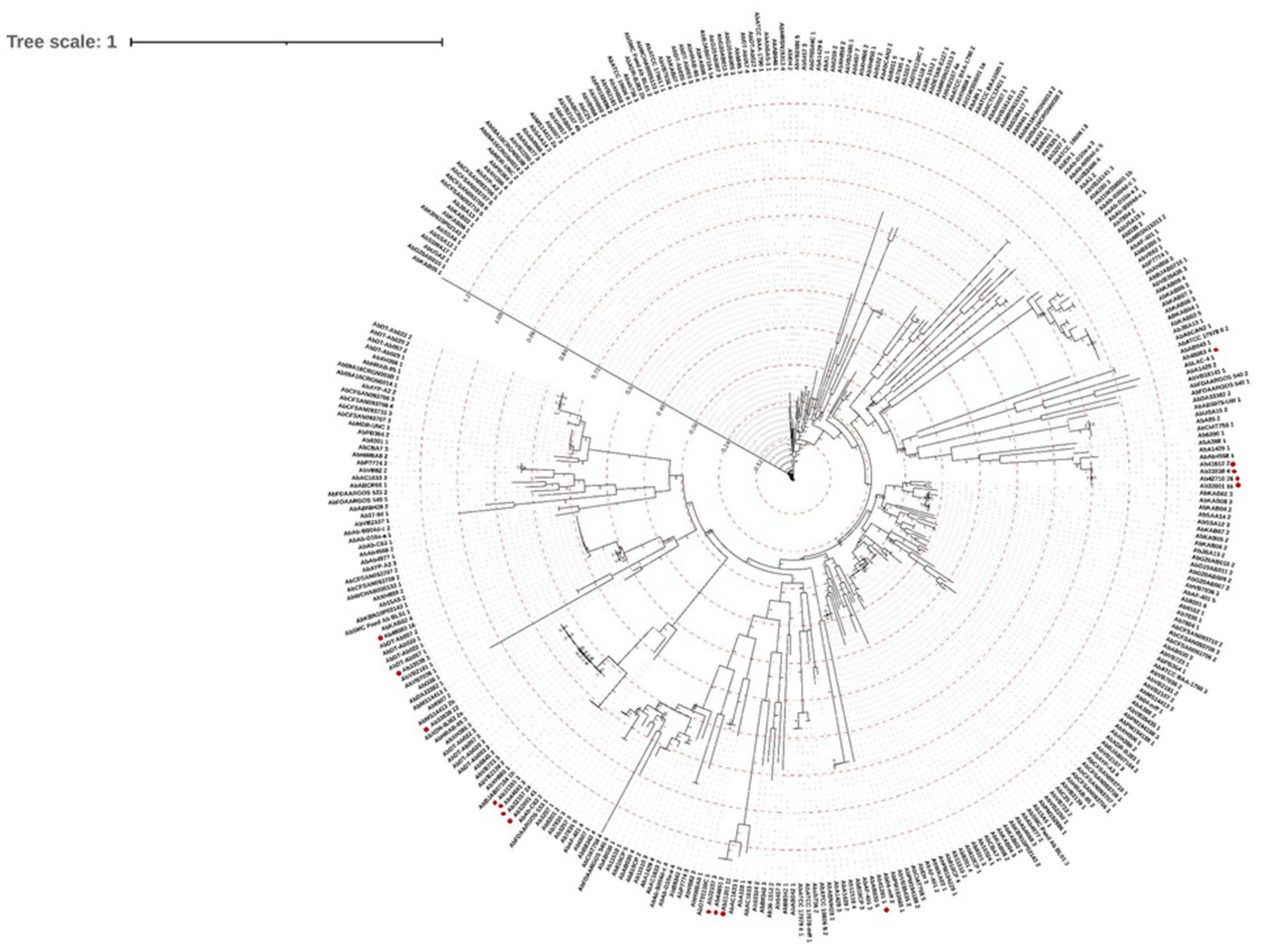
Prophage genome phylogenetic tree. A maximum likelihood phylogenetic tree was constructed based on the alignment of 348 nucleotide sequences of complete prophages, using the FastTreeMP 2.1.11 tool (Price et al., 2010). The tree was analyzed and annotated using the Interactive Tree of Life (iTOL) v6 (Letunic and Bork, 2019). Red circles highlight prophages identified in the newly sequenced genomes.

The nucleotide sequences of the 348 complete prophages were aligned by MAFFT and a maximum likelihood phylogenetic tree was constructed (Figure 4), revealing high diversity. Notably, prophages identified in newly sequenced genomes from Portugal, tend to cluster together into three distinct groups. Our analysis further revealed that, while prophages within these local genomes exhibit greater similarities with each other, similar counterparts were also found in genomes sequenced from strains isolated in other locations.

**Figure 5:**
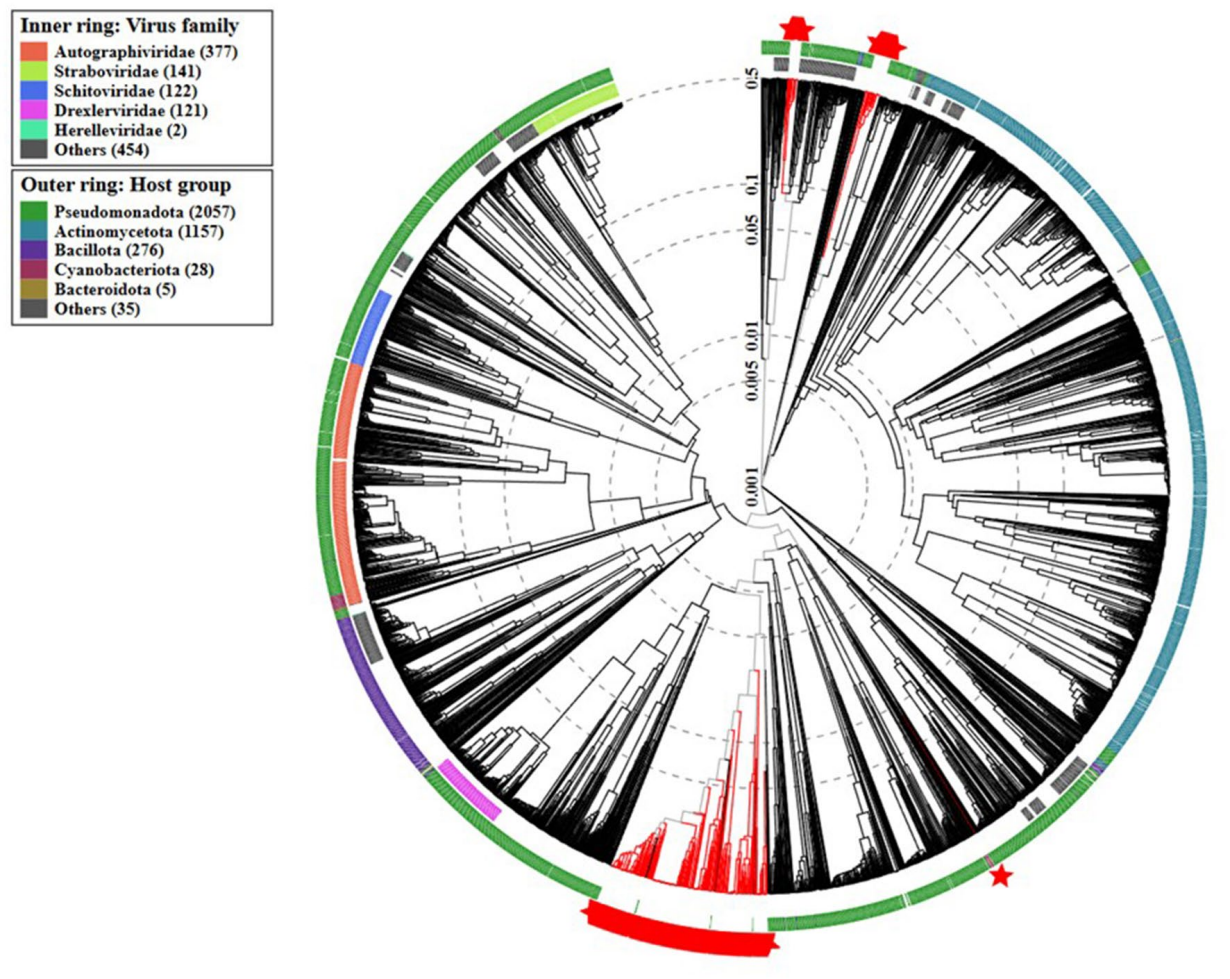
Proteomic tree generated by VipTree version 4.0 (Nishimura et al., 2017) showing the phylogenetic placement of the analyzed complete prophage (displayed in red) sequences within the viral taxonomy.

The proteomic analysis performed using VipTree (Figure 5) revealed that the prophage sequences identified in *A. baumannii* genomes clustered closely with several phages of *Burkholderia* and some of *Pseudomonas* genus. Notably, our group of prophages clustered with previously described *Acinetobacter* phages.

Moving into genomically coherent families better reflects the diversity and genomic relationships of these prophages, allowing for more accurate assessments of their evolutionary history and functional potential (Turner et al., 2023). In this regard, we utilized the taxMyPhage webserver (Millard et al., 2024), a new tool which was designed for the taxonomic classification of prophage sequences. Out of the 348 sequences analyzed, only 18 (5.17%) were successfully classified at the genus level, all of which were assigned to the genus *Vieuvirus*. One of these sequences was classified at the species level as *Vieuvirus* B1251 (Supplementary Table S1). *Vieuvirus* genus belongs to *Caudoviricetes* class, Uroviricota phylum, Heunggongvirae kingdom and Duplodnaviria realm.

### 3.5 Putative lysins identification

**Figure 6:**
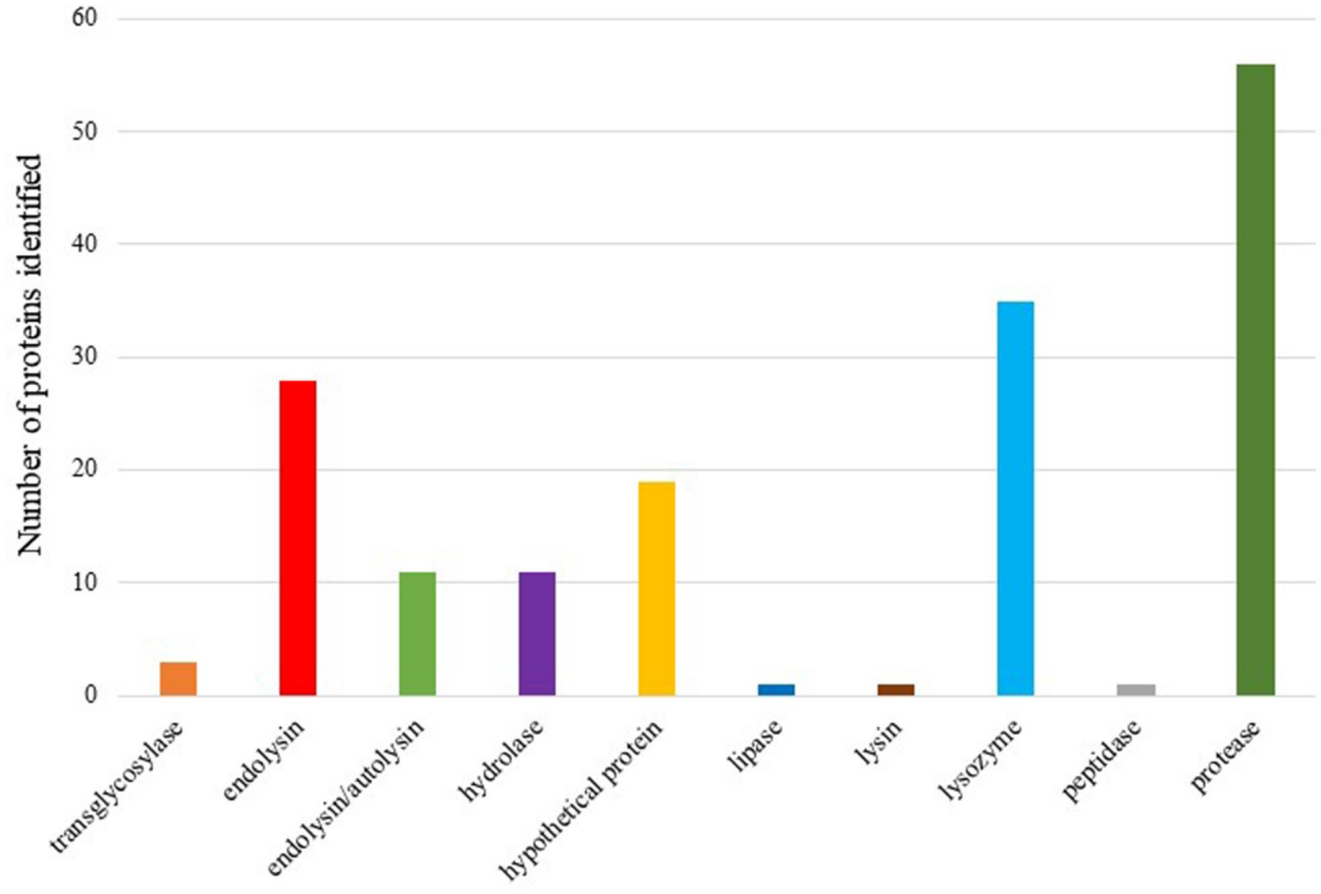
Bars graph showing putative lysins present on the complete prophages analyzed.

Due to the terminology diversity, keywords were searched for proteins related to lysis in complete prophage regions. The initial set of proteins was analyzed based on the homology of the sequences by aligning the amino acid sequences against databases, which allows us to acquire some information about the function of the proteins. BLASTp results enabled to keep 166 proteins under study that were indicated as having homology with proteins whose function was related to the lysis of the bacterial cell. To facilitate the interpretation of the data, 12 categories were established: cell wall hydrolase, lysozyme, endolysin/autolysin, peptidase, lysin, protease, hydrolase, tail lysozyme, hypothetical protein, transglycosylase, lipase and endolysin: we could identify putative lysins in 10 out of the 12 categories (Figure 6). Putative lysins from all categories have been observed in uncertain prophage regions.

## 4 Discussion

Infections caused by *A. baumannii*, particularly in hospital settings, represent a significant challenge due to the increasing emergence of resistance to available antibiotics. Given this context, the study of *A. baumannii* prophages is vital as they represent a promising source of antimicrobial agents for phage therapy. Notably, proteins encoded by these prophages, such as lysins, have the potential to target and disrupt bacterial cell walls, providing alternative therapeutic avenues against resistant strains. Additionally, lysins have a low likelihood of resistance development due to their dependence on relatively conserved peptidoglycans, making them a reliable option for treatment. Furthermore, they demonstrate synergistic effects when combined with other lysins or antibiotics, enhancing their efficacy against pathogens that colonize mucosal surfaces and form biofilms (Oliveira et al., 2013; Czaplewski et al., 2016; Lin et al., 2017).

In our study, we observed a widespread of prophages in *A. baumannii*, with some strains harboring multiple prophage regions. This finding aligns with previous research indicating that many *A. baumannii* strains are polylisogenic (Badawy et al., 2020). The presence of complete prophages suggests recent integration events, which may enhance our understanding of *A. baumannii* evolution and adaptability. Complete prophage, found to be of medium or high-quality completeness, have preferred integrations sites within *A. baumannii* genomes, which may be related with the specificity of the encoded integrase. The presence of numerous uncertain prophage regions, presumably prophage remnants, might be expected due to the strong selective pressures that lead to the degradation or excision of integrated prophages (Bailey et al., 2024; Vale et al., 2024).

Prophages play a significant role in bacterial genome evolution, as they serve as vehicles for genetic diversification and expansion (Vale et al., 2022). We have found that the bacterial genome size excluding prophage elements remains relatively constant, while the insertion of prophages leads to substantial genomic growth. This observation suggests that prophages are the primary contributors to the increase in genome size, thereby playing a critical role in shaping the genomic architecture of *A. baumannii.*

Bacteriophages have high genetic diversity, which makes it difficult to identify homologous proteins and compare the structural modules that generate them (head, neck, tail). That presents a challenge to establish a classification of the phages (Lopes et al., 2014), particularly after the recent revision of the ICTV taxonomy that abolished morphotypes. However, it is important to note that the current taxonomic classification of prophages remains a challenge due to the limitations of the new ICTV taxonomy, which often does not determine their exact classification, indicating that they may belong to novel, yet-to-be-classified families within the class *Caudoviricetes*. While a small proportion of sequences were classified at the genus level, the majority of prophages could not be given a complete taxonomic assignment, reflecting the high genetic diversity of phages and the ongoing refinement of viral taxonomy.

The maximum likelihood phylogenetic tree (Figure 4) evidences a high genomic prophage variability. However, the clustering of the prophages from the newly sequenced genomes in Portugal suggests that they share evolutionary relationships, possibly indicating the presence of region-specific or locally adapted phage populations. This pattern may reflect similar ecological pressures, horizontal gene transfer events, or shared hosts within the Portuguese environment, highlighting how local bacterial populations may acquire and maintain prophages with related genetic characteristics. Importantly, the identification of similar prophages in genomes from strains isolated in other regions suggests that, while local prophage populations may exhibit some degree of region-specific adaptation, there is also potential for broader, generalized applications of these prophages in phage-based therapies across different geographies. Furthermore, while complete prophages tend to cluster together within their respective families, exceptions exist that point to the complexity of bacteriophage evolution. This emphasizes the necessity for genomic-based approaches in phage classification to complement traditional morphological methods. Within a broader viral taxonomy context, the proteomic tree showed that our prophages clustered mainly with *Burkholderia* and *Pseudomonas* phages.

Bacteriophages are currently classified into genera and species based on genomic similarity, requiring comparisons with all phage genomes defined by the ICTV (Millard et al., 2024). Notably, the phage taxonomy is still under development and remains incomplete, as many prophage sequences lack clear taxonomic resolution. This is particularly evident considering the large number of sequences that could not be classified further than the class *Caudoviricetes* (94.83%), reflecting the gaps in the current phage databases and the need for further refinement of taxonomic frameworks. The ones that were possible to classify were of the *Vieuvirus* genus, which was also found to be the taxonomical genus of the previously described *Acinetobacter* phages that clustered with our prophages in the proteomic tree. This observation reinforces the classification that we obtained.

Prophages have coexisted and evolved alongside bacteria, developing highly effective mechanisms to lyse and destroy their bacterial hosts at the end of the lytic cycle, facilitating the release of viral progeny. Lysins are proteins involved in this lysis process and have been widely researched and applied in the production of new antibacterial treatments (Sykilinda et al., 2018; Antonova et al., 2019; Kim et al., 2020). Our investigation into lysins revealed a rich diversity within the prophages analyzed. We identified 166 proteins potentially involved in lysis across complete prophages. Among these, various types of lysins were detected, underscoring the potential of these proteins as therapeutic agents against *A. baumannii*. The high number of prophage regions encoding a diverse array of lysins suggests that these could serve as a valuable resource for developing novel antibacterial treatments. Future work will focus on cloning and producing the identified lysins, followed by evaluating their antimicrobial activity. Such efforts hold promise for advancing therapeutic options against infections caused by antibiotic-resistant *A. baumannii*, contributing to the broader fight against MDR pathogens.

In conclusion, the insights gained from this study emphasize the intricate relationship between prophages and their bacterial hosts, the potential of lysins as antimicrobial agents, and the importance of refining phage taxonomy to reflect the complexities of viral diversity and evolution.

## Data Availability

The next generation sequencing reads were submitted to the Sequence Read Archive (SRA), with the accession numbers ERS21116715-ERS21116722, ERS21116724, and ERS21116725 for *A. baumannii* isolates from the collection of the National Institute of Health Dr. Ricardo Jorge, and ERS21116705-ERS21116713 for the *A. baumannii* isolates of the collection of the Faculty of Pharmacy of the University of Lisbon.

## Footnotes

^1^ https://www.bv-brc.org/

^2^ https://www.geneious.com
